# Functional Data Analysis for Predicting Pediatric Failure to Complete Ten Brief Exercise Bouts

**DOI:** 10.1109/JBHI.2022.3206100

**Published:** 2022-12-07

**Authors:** Nicholas Coronato, Donald E. Brown, Yash Sharma, Ronen Bar-Yoseph, Shlomit Radom-Aizik, Dan M. Cooper

**Affiliations:** US Military Academy, West Point, NY 10996 USA; University of Virginia, Charlottesville, VA 22904 USA; University of Virginia, Charlottesville, VA 22904 USA; University of Virginia, Charlottesville, VA 22904 USA; University of California, Irvine, CA 92697 USA; University of California, Irvine, CA 92697 USA; University of California, Irvine, CA 92697 USA

**Keywords:** Machine learning, generalized spectral additive models, time series, cardiopulmonary exercise testing, CPET

## Abstract

Physiological response to physical exercise through analysis of cardiopulmonary measurements has been shown to be predictive of a variety of diseases. Nonetheless, the clinical use of exercise testing remains limited because interpretation of test results requires experience and specialized training. Additionally, until this work no methods have identified which dynamic gas exchange or heart rate responses influence an individual’s decision to start or stop physical activity. This research examines the use of advanced machine learning methods to predict completion of a test consisting of multiple exercise bouts by a group of healthy children and adolescents. All participants could complete the ten bouts at low or moderate-intensity work rates, however, when the bout work rates were high-intensity, 50% refused to begin the subsequent exercise bout before all ten bouts had been completed (task failure). We explored machine learning strategies to model the relationship between the physiological time series, the participant’s anthropometric variables, and the binary outcome variable indicating whether the participant completed the test. The best performing model, a generalized spectral additive model with functional and scalar covariates, achieved 93.6% classification accuracy and an F1 score of 93.5%. Additionally, functional analysis of variance testing showed that participants in the ‘failed’ and ‘success’ groups have significantly different functional means in three signals: heart rate, oxygen uptake rate, and carbon dioxide uptake rate. Overall, these results show the capability of functional data analysis with generalized spectral additive models to identify key differences in the exercise-induced responses of participants in multiple bout exercise testing.

## I. INTRODUCTION

The human cardiovascular and associated systems are dynamic and highly interrelated. A major goal of cardiopulmonary exercise testing (CPET or CPX) is to identify physiological variables using nondestructive and minimally invasive protocols that enable the clinician or researcher to make predictions about an individual’s particular condition or level of physical fitness. Standard exercise testing procedures produce outputs that must be interpreted by trained practitioners with an understanding of the underlying physiology and kinetics of the system, as well as an ability to interpret multiple time series. By applying machine learning (ML) techniques to multiple bout exercise testing, we seek to lay the foundation for quicker and more consistent interpretation of patterns in physiological time series that may aid researchers in caring for their patient. Our work with functional data analysis shows that it may be a highly useful method for classifying patients based on their exercise-induced cardiovascular signals.

In typical CPET protocols the work performed becomes increasingly difficult until the participant or technical supervisors sense that the limit of the individual’s tolerance has been reached. In contrast to most CPET protocols, patterns of physical activity in children and adolescents observed outside of the laboratory are characterized by series of brief bouts of exercise of varying intensity interspersed with short intervals of rest [1], [2], [3]. Consequently, individuals must frequently decide whether to begin the next bout of exercise when engaged in these more natural patterns of sporadic physical activity. We wondered whether we could identify predictive physiological signals from breath-by-breath gas exchange and heart rate (HR) data that are collected in CPET laboratories.

The study has two innovations: First, we show that systematically processing the time series with Functional Data Analysis can lead to conclusive predictive results for pediatric participants. Second, we posit that an alternative exercise test (MBEB) may be more appropriate for children and provide richer results than the gold-standard maximal effort CPET test.

The next section provides relevant background and a literature review for this area of research, which led us to develop the research questions defined in Section II. To address these research questions we obtained exercise test data from 81 participants. We then applied Functional Data Analysis to characterize the multiple time series obtained from the exercise testing. Results from this analysis are in Section IV. Following a discussion of results and their implications (Section V), we provide an overview of potential future research opportunities (Section VI) and limitations of our study (Section VII).

## II. BACKGROUND

### A. Medical Interpretation of Exercise Testing Data

Exercise testing for diagnostic purposes is conducted by measuring physiological responses during graded physical exercise. Typically, this is done by measuring gas exchange and cardiac condition in order to score the performance of coordinated human biological subsystems. CPET is the most widely used exercise test; it measures responsiveness of the pulmonary, cardiovascular, neuropsychological, skeletal muscular, andhematopoieticsystems. Since about the 1920s, peak oxygen uptake $\left(\dot{V}O_{2peak}\right)$ has been the most widely used biomarker for aerobic fitness, commonly measured through CPET protocols as the “gold-standard” [4]. CPET has the advantage of being low-risk [5] and non-invasive.

The voluminous data from continuous electrocardiogram and other measurements during CPET are described in a 9-panel report which can be leveraged for clinical diagnosis of cardiopulmonary abnormalities [6]. Armed with the ability to analyze CPET reports, medical professionals should be able to definitively diagnose – or predict – these abnormalities. However, there exist numerous limitations to CPET interpretation and it is reportedly underutilized as a clinical tool [7], [8], [9], [10], [11], [12].

A survey of recent literature reveals an effort to overcome the barriers to CPET interpretation. To address the difficulty with human interpretation of graphical data and chaotic breathing patterns, [7] recommends data smoothing techniques as well as a focus on dynamic measurement relationships indicating patterns of dysfunction. Our approach optimally smooths the data and draws out underlying functional relationships to assist in pediatric fitness assessment.

In [8] analyzed heart failure severity using time series data and statistical analysis of variance to compare their engineered variables. They aimed to clarify pathophysiology with a single display that uses ratios of oxygen uptake, ventilation, and carbon dioxide output, plotted on equal axes, to better quantify heart failure severity. In [9] notes that when processing multiple CPET time series data points, we often simplify peak values and slopes which leads to a loss of valuable trend information. The authors propose a method for encoding the CPET time series as images, which are fed to a convolutional neural network to classify patients. In this work, our method is directly compared to the performance of the image-encoding approach.

Computer-aided algorithms in [10] were highly useful in evaluating CPET data to identify medical conditions. This experiment with incremental exercise tests produced data that, when input to supervised ML algorithms, helped to discriminate between healthy and diseased patients (mean accuracy 99%). The novel contribution was to convert raw CPET data into ‘normalized percent of predicted’ values.

Recently, [11] applied CPET-generated data to aid clinical evaluation of exercise intolerance. This advanced approach involved feature engineering, feature selection, and automatic ML classification to choose the best-performing model for 225 CPET time series cases. In [11] also calls for further investigation as to whether early data capture would facilitate accurate diagnosis without the need for maximal-effort CPET. Our work addresses this research gap; our results suggest that data from exercise tests of shorter duration can be incredibly useful in understanding an individual’s fitness status.

### B. Beyond CPET

Though CPET has long been the gold standard, some researchers have been investigating other, possibly more effective ways to capture health and fitness information in pediatric patients. References [1], [2], [3] propose that an alternative to CPET could be more suitable for younger populations. Among other key differences, it has been noted that gas exchange and ventilatory signals tend to show greater variation in children than adults [13]. The present study utilizes a protocol termed “Multiple Brief Exercise Bouts” (MBEB) which follows the reasoning that natural patterns of physical activity in children are characterized by relatively short bursts (seconds to minutes) of exercise at various intensities interspersed with rest. By observing the same gas exchange and frequency variables as CPET over a more appropriate fitness test protocol, we hope to glean important physiological insights about square wave exercise cardiovascular dynamics in pediatric subjects.

In a recent publication, we analyzed the gas exchange and HR kinetic responses during the first five bouts of MBEB and compared data from early and late-pubertal females and males at low- and high-intensity MBEB [3]. In the course of these studies, we noted that all participants completed the MBEB task when the MBEB work rates were low intensity. In contrast, during the high-intensity MBEB, a significant number of participants were unable to start the next bout after the 1-min rest. An important finding of the research was that, during high-intensity MBEB, the dynamics of HR and gas exchange changed from bout to bout even though the work rate input remained constant. This result suggests that recovery from each bout was incomplete and raises the possibility that the cumulative response deficiency might eventually translate into signals that alter cognitive exercise behavior. In this research, we present an analysis of gas exchange and HR data in the bouts preceding the task failure.

When reviewing the literature, we found no similar application of Functional Data Analysis (FDA) to CPET time series. This paper inspires a deeper look into FDA as a viable approach to processing multiple bout exercise data. Our study was guided by two primary research questions:

1. Can we use machine learning techniques and FDA to accurately predict which individuals will fail to complete an exercise test based solely on their cardio-respiratory signals? Can we make this prediction with reasonable accuracy after only four exercise bouts?
2. To what extent do the machine learning techniques use sex, maturational status, and body mass to predict the physiological responses of children during MBEB?

Shorter and simpler exercise tests would be hugely beneficial to the medical community; thus, we sought to make predictions on just 720 seconds of MBEB.

We decided to study the effects of sex, puberty, and body mass as they are readily available in most CPET datasets and are known contributors to physiological responses during exercise. We were interested to explore to what extent each of the three anthropometric covariates aided with prediction and, presumably, impacted the child’s exercise tolerance threshold.

## III. METHODS

### A. Study Participants

Human data collection was approved by University of California at Irvine’s IRB HS# 2015-2467. Eighty-one participants were recruited, of which seventy-eight were included in the final analysis. As shown in Table I, the participants were equally distributed. The volunteers reflected the racial and ethnic composition of the region [Caucasian (93%), Hispanic/Latino (5%), and African American (2%)]. All participants were screened and determined to be healthy based on interviews to identify any congenital or chronic conditions that would impair physiological responses to exercise. Extremely physically active participants (e.g., elite athletes involved in routine intensive exercise training) were also excluded. A commonly used self-assessment questionnaire for population studies was used to assess pubertal status, quantified as early pubertal (Tanner 1–2) and late pubertal (Tanner 4–5).

### B. Collection of Exercise Testing Data

The study consisted of three separate exercise testing sessions completed over a course of no more than 12 weeks. Study visits were scheduled to morning or early afternoon and participants were asked to abstain from exercise before the visit in the same day. The first session consisted of a ramp-type progressive exercise test in which the participant pedaled on a cycle ergometer (CE) until they reached the limit of tolerance. Gas exchange was measured breath-by-breath using the SensorMedics system (Vmax Encore 229, Yorba Linda, CA). Participants were vigorously encouraged to continue pedaling during the high-intensity phases of the test. Gas exchange was measured breath-by-breath and peak $\dot{V}O_{2}$ was determined when the respiratory exchange rate exceeded 1.0 and was calculated as the highest 20-s rolling average in the last minute of exercise.

The results of the ramp CPET were then used to set the individualized baseline work rate for the subsequent MBEB session scheduled for separate days. The work rate for the MBEB task was calculated for each participant as low-intensity (40% of peak work rate) and high-intensity (80% of peak work rate). The MBEB protocols were performed on different days and in random order. No warm-up exercise was performed.

MBEB consisted of up to ten 2-min bouts of constant work rate exercise on a CE with a 1-min rest period after each bout. After each bout, the participants were instructed to affirm their willingness to continue with the next bout. For all sessions, we asked each participant to try to complete ten bouts of exercise. All participants completed the full 10-bout MBEB task at the low-intensity work rate; these data were not analyzed. In contrast, 39 of the 81 participants (48%) failed to complete the high-intensity MBEB. This group of ‘task-failed’ participants completed a mean of 6.18 ± 0.23 bouts and all 39 completed at least 4 bouts. After time-interpolation to achieve second-by-second data for every participant, the final data set consisted of 266,416 discrete observations of anthropometric, frequency, and gas exchange variables measured at high intensity.

### C. Functional Data Analysis

As a tailorable exercise protocol, MBEB produces data in discrete but sometimes irregular time series. Variability in the intervals of measurement and correlation of repeated measurements are just two of the potential problems that arise with MBEB output that present challenges for traditional multivariate statistical techniques. Since the goals of this study are to provide a high level of classification accuracy and to present readily interpretable and physiologically relevant results for clinicians, we need methods that can address these challenges. The complexity of traditional multivariate models can render their analysis uninformative to the medical community. Additionally, we hypothesize that a high amount of understandable information can be gleaned from exercise test data without a traditional 9-panel CPET plot. The systematic method we use to attain these goals and address the data challenges is Functional Data Analysis (FDA).

FDA is a highly flexible technique which can deal with non-independent and correlated repeated measures. Its prominence has grown simultaneously with the emergence of electronic devices that accurately capture a continuous stream of physiological data; FDA can help leverage that data towards meaningful empirical conclusions.

Within a biomedical context FDA has proven powerful in the analysis of human growth curves [14], gait analysis [15], fetal heart rate monitoring [16], [17], and prediction of maximal $\dot{V}O_{2}$ during exercise [18]. Additionally, [18] proposed FDA to reduce predictive error in estimation of maximum HR by avoiding the problems of high dimensionality and collinearity. A ramp exercise protocol was used in that research, and the authors called for exploration into the predictive capacity of FDA with square wave exercise modalities. Our research applies MBEB as a square wave modality.

When implementing FDA, data observations do not need to be equally spaced and missing observations are handled relatively well. Exceptionally noisy signals (such as respiratory rate in our data) benefit from the smoothing procedure, which is the key first step in FDA. The functional data (FD) objects themselves can be more visually informative than the set of finite discrete observations and allow us to draw prediction information by applying multivariate statistical concepts.

The functional nature of MBEB-derived observations encourages us to assume that the data are realizations of stochastic processes in continuous time. The time series measurements of our MBEB experiment are discrete and sometimes noisy observations of a continuous, dynamic process, therefore FDA seems highly appropriate. After transforming the breath-by-breath or second-by-second time series into a collection of smooth FD curves, we can explore supervised or unsupervised ML techniques.

#### 1) Data Conversion Procedure:

The first step of FDA was to convert the raw time series into FD objects by choosing the appropriate basis transform and smoothing parameters. To predict ‘task-failures,’ we included only measurements for the first 720 seconds of MBEB. The purpose was to analyze only the first four bouts of MBEB, as all 81 participants completed a minimum of four bouts.

**Fig. 1.**
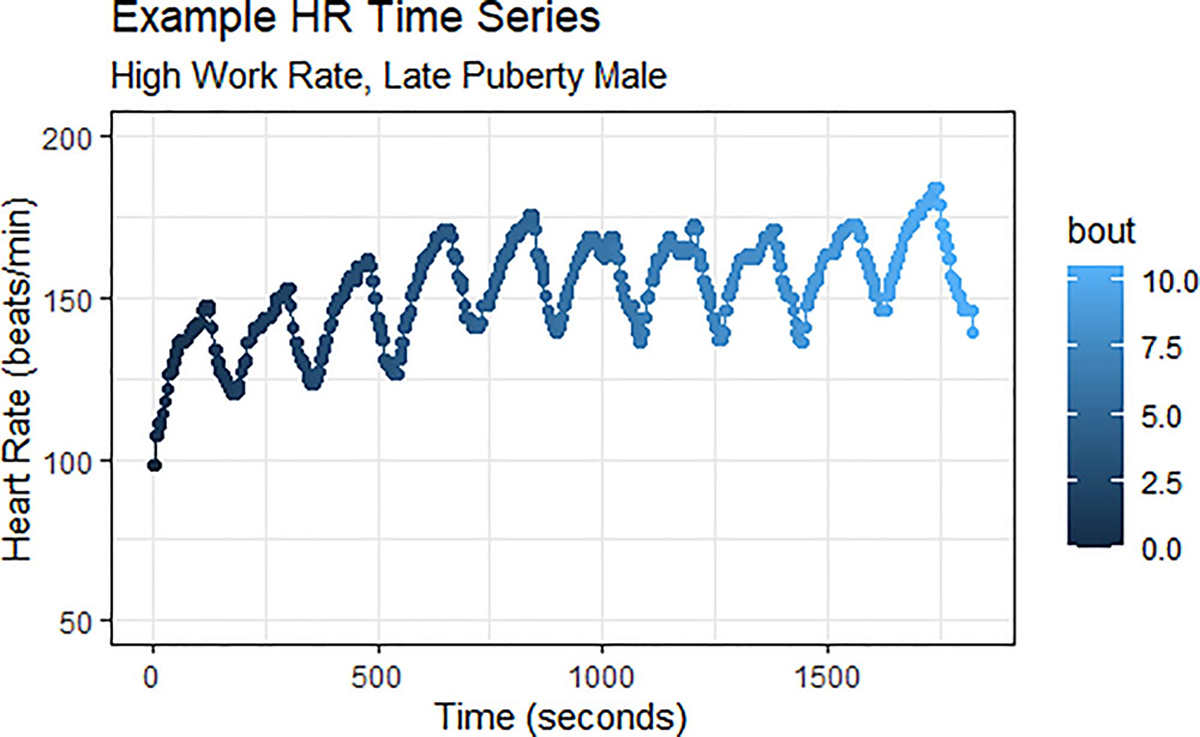
One participant’s second-by-second heart rate for the full MBEB session. In general, HR was the signal that contained the least noise in our data set; individual exercise bouts are very easily discerned.

**Fig. 2.**
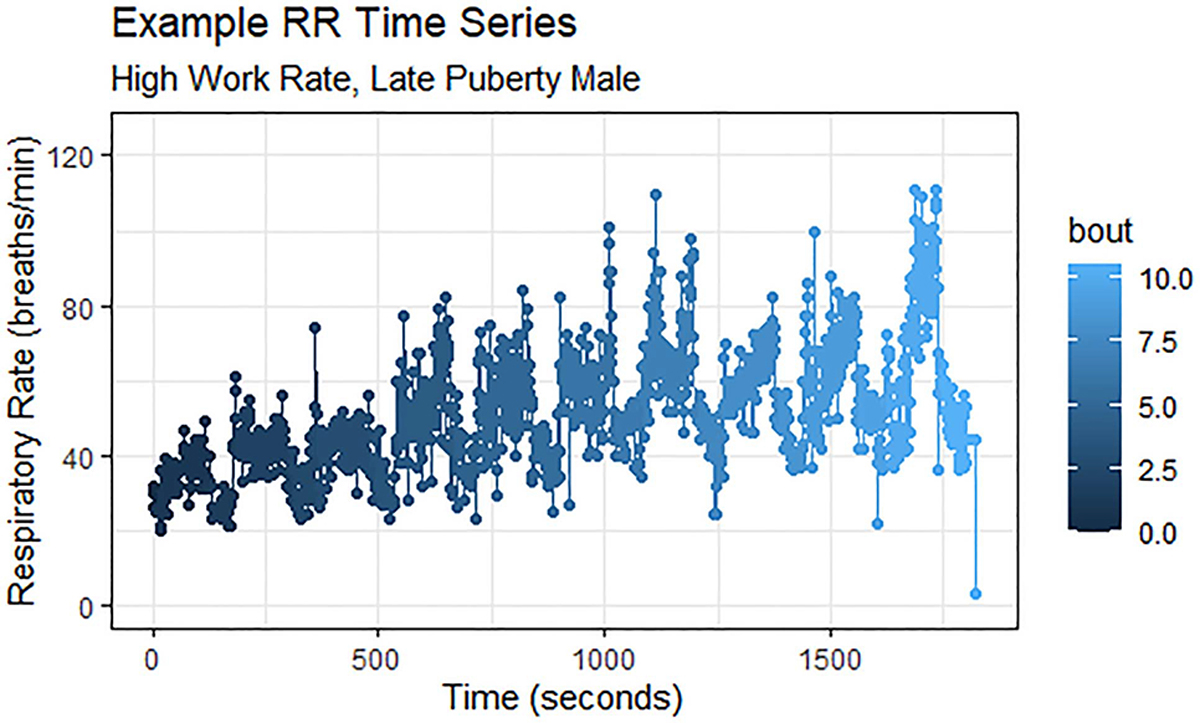
One participant’s second-by-second respiratory rate for the full MBEB session. In general, RR was the signal that contained the most noise in our data set; individual exercise bouts are difficult to discern.

The four variables of interest for our research question were heart rate (HR) (beats/min), respiratory rate (RR) (breaths/min), $\dot{V}O_{2}$(mL/min), and $\dot{V}CO_{2}$ (mL/min). The data was organized such that each response variable constituted its own independent time series. We confirmed that each signal has a distinct pattern characterized by variation and noise. Sample representations of two signals are presented in Figs. 1 and 2. Plots of the full data set are available in Appendix A.

**Fig. 3.**
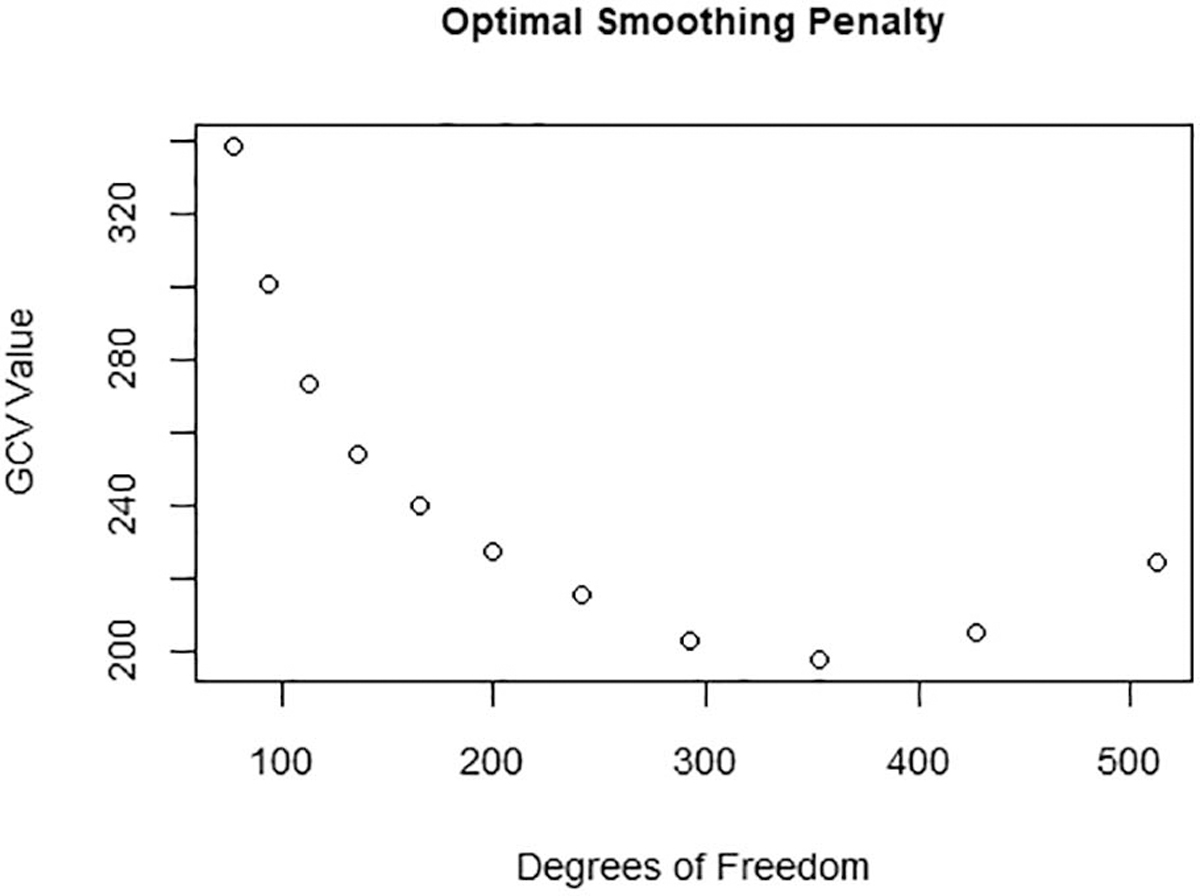
Example estimation of the smoothing parameter λ. An appropriate level of smoothing was determined by visual inspection of the relationship between GCV and DoF in the smoothed model. This procedure is explained in depth in [21]. This figure shows a minimal GCV when the model contains 350 DoF, which corresponds to a λ near 200. Thus, 200 was chosen as the smoothing penalty for the set of HR curves, and the fit was validated after visual inspection of the smoothness (see Fig. 4). This process was repeated for all variables.

Smoothing the data helps our algorithm to differentiate normal breath-by-breath noise from signal patterns indicating that a participant is reaching their exertion limit. Splines have been chosen to represent similar time series data in previous studies [19], [20]. A B-spline basis representation was determined to provide an excellent fit to each of the four time series. The splines were generated using 725 total basis elements of 6th order B-splines. The smoothing procedure was controlled by a roughness penalty, which resulted in reasonably smooth functions without unacceptably large variations in the approximating function. Penalized smoothing was done by applying harmonic acceleration operators to the functional data and searching across values of λ (smoothing parameter) until an acceptable generalized cross-validation (GCV) error level and degrees-of-freedom (DoF) were reached in the smoothed estimate. In other words, each of the response curve sets were deemed appropriately smooth for this particular application. This process is introduced in Chapter 5 of [21]. Fig. 3 explains this procedure visually.

Three participants were removed due to irregularities in their time series (likely the result of technical HR or gas exchange data collection errors). This left 78 curves for analysis, representing an equal number of task-failed (n = 39) and task-successful (n = 39) participants.

**Fig. 4.**
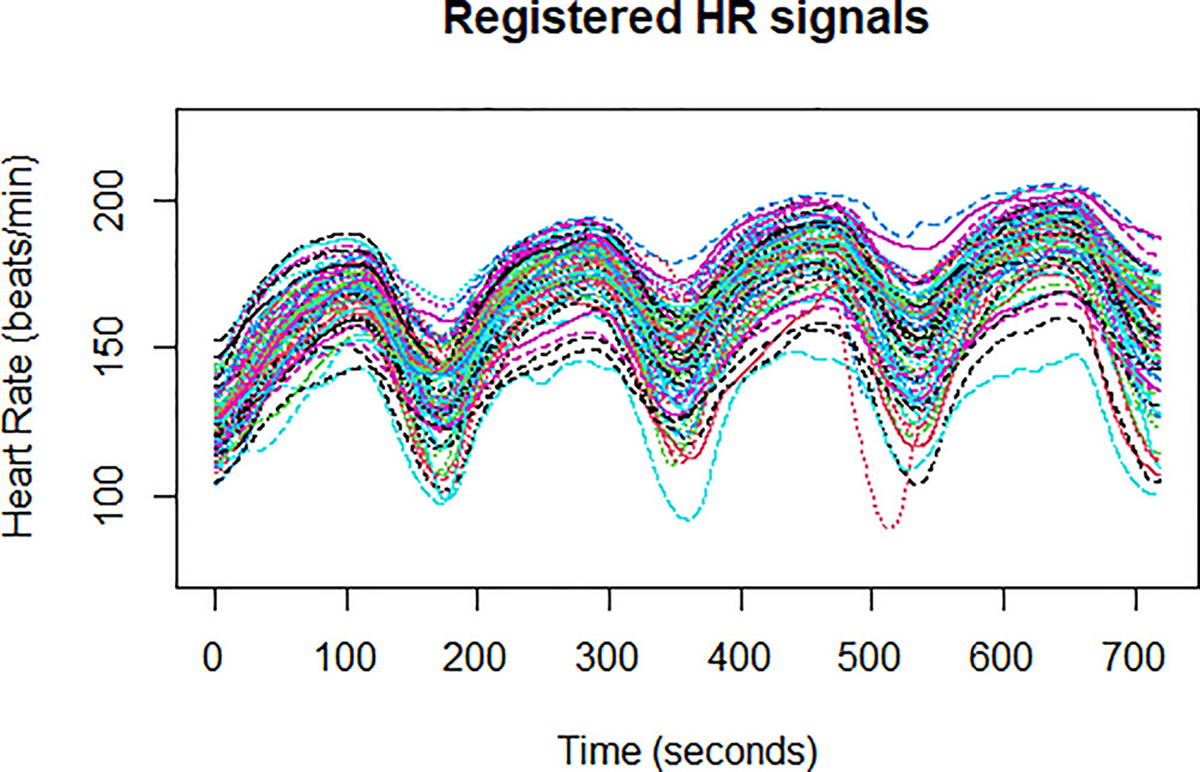
Heart Rate data after converting the discrete time series to 78 smoothed and registered curves. Each participant’s time series is represented as an individually colored function.

After smoothing the FD objects, we carried out curve registration which allows us to align the curves (by time warping or otherwise) and remove phase variation while maintaining amplitude variation. In [22] presents the foundation for registration in misaligned data sets. For our data, automated continuous registration resulted in minimal phase shifting, as the original time series were nearly perfectly aligned by nature of the testing protocol; exercise bouts began and terminated near the same moment in time for all participants. The proportion of total variation due to phase variation (*MSE*_*phase*_*/MSE*_*total*_) was 9%. The registered curves (with phase variance removed) were utilized for all subsequent analysis. Fig. 4 depicts the smoothed and registered HR FD objects as an example; remaining plots are in Appendix B.

#### 2) Functional t-Tests:

**Fig. 5.**
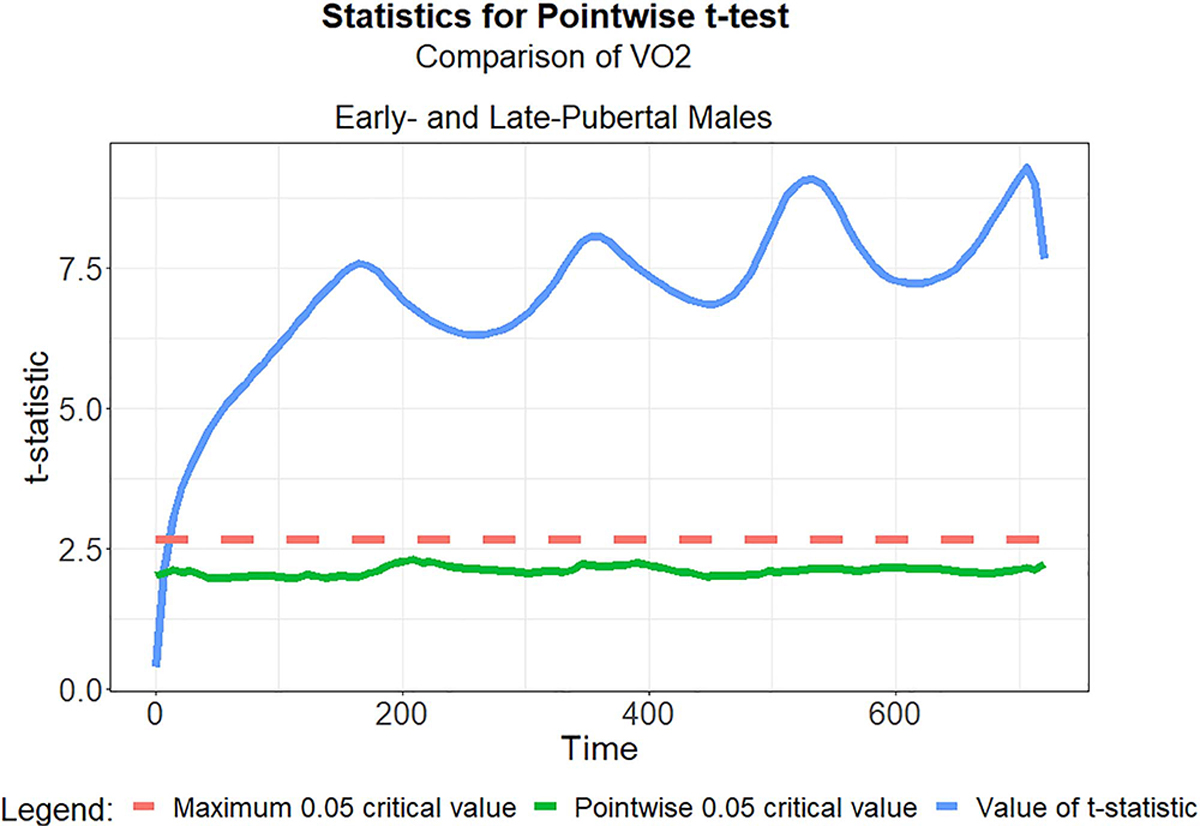
Visual output of the functional permutation *t*-test between Early- and Late-puberty males. The blue curve shows the *t*-statistic for the observed values. The green curve represents the 95% quantiles, and the dashed red line is the 95% quantile of the maximum of null distribution *t*-statistics. The *t*-test confirms that the derivatives are indeed different except in the regions of overlap (the first few moments of exercise). This could signify a fundamental difference in the physiology between puberty groups when holding gender status constant.

To identify differences in gender or puberty sub-groups, we investigated the null hypothesis (*H*_0_) that there exists no statistically significant difference in the functional means of participants in contrasting sub-groups. To test for differences between *gender groups*, we sampled 11 each of males and females at the early-puberty level, to which we applied permutation *t*-tests on their functional means using the default parameters of the function *‘tperm.fd’* in the R software package *‘fda’* [23]. To test for differences between *puberty groups*, we compared 11 samples of early puberty males vs. late puberty males. For time periods where the *t*-statistic exceeded the critical value (0.05), we could reject (*H*_0_). This procedure revealed distinct puberty and gender differences throughout the time series of $\dot{V}O_{2}$ and $\dot{V}CO_{2}$, but no such significant mean functional difference existed for HR and RR. Fig. 5 highlights one result of this exploration.

#### 3) Supervised Functional Classification:

We tested the ability of the FDA approach to discriminate between MBEB task-failures and task-completers. This is an example of a curve-discrimination problem, further explored in [24]; we have a sample of curves (*X*_*i*_, *i* = 1, …, *n*), and each of them is known to belong to one of the *G* groups *g* = 1, …, *G*. Given a new curve *x*, we wish to know its class membership; thus we estimate, for any *g* ∈ 1, …, *G*, the conditional probability: *p*_*g*_(*x*) = *P*[*T* = *g* | *X* = *x*] where *T*_*i*_ is the group of the curve *X*_*i*_ (task-failure or completer). To do this we applied various classification models to the FD object set. The goal was to find a classifier with the minimum error rate. Our first approach was to predict ‘failure’ from combinations of the functional data and anthropometric covariates: sex (binary), puberty level (binary), and body mass (continuous). Weight alone was used for body mass observations, without consideration to fat mass.

The flexible nature of FDA allowed us to test seven unique classification models: generalized spectral additive models (GSAM), linear discriminant analysis (LDA), recursive partitioning and regression trees (RPART), RandomForest (RF), support vector machines (SVM), neural network (NNet), and *k*-Nearest Neighbors (KNN). Ten-fold cross-validation was built into each classification model.

Functional representation of HR alone was the first FD covariate we tested: *failure* = *s*(*HR*_[0,720]_) where *HR*_[0,720]_ is the smoothed HR function over the first four bouts. After this approach proved fruitful on the cleanest physiological signal, we applied the classifiers to RR, $\dot{V}O_{2}$, and $\dot{V}CO_{2}$ FD objects with the same model parameters. This allowed us to compute model performance and directly compare results. Overall model accuracy was calculated as the number of correct classifications divided by the total number of attempts. The F1 score was computed as (2 ∗ (*precision* ∗ *recall*)*/*(*precision* + *recall*)). Finally, we combined all anthropometric and functional covariates for HR, RR, $\dot{V}O_{2}$, and $\dot{V}CO_{2}$ into a ‘full’ multivariate model and tested the classification rate. The structure of each model is described in Appendix D.

The final step was to conduct functional analysis of variance (FANOVA) over our 78 independent samples. One-way ANOVA was performed within the software package *‘fda.usc’* based on an asymptotic version of the ANOVA F-test. The function returns the *p*-value of the test over a specified number of bootstrap replications [25]. The HR, RR, $\dot{V}O_{2}$, and $\dot{V}CO_{2}$ functional data objects were bootstrap resampled 500 times, plotted, and analyzed. We empirically tested whether ‘task-failures’ and ‘task-completers’ display differences in their signals’ functional means, as indicated by a significant FANOVA *p*-value. A *p*-value value ≤ 0.05 was considered significant to reject the null hypothesis (*H*_0_) of equality of mean functions between participants labelled ‘failure’ and ‘completer.’

#### 4) Comparison:

We compared our FDA method to the recently proposed image encoding approach for CPET classification [9]. In that paper, authors encoded the CPET time series as images using the Gramian Angular Field (GAF) or the Markov Transition field (MTF) approach, followed by attention-based pooling for multivariate time series classification. GAF/MTF encoded images are capable of capturing the temporal trends and interactions between different time points within time series and hence have shown strong classification performance. We encoded the time series used in our ‘full’ multivariate model (HR, RR, $\dot{V}O_{2}$, and $\dot{V}CO_{2}$) using the three approaches (GASF, GADF, and MTF) proposed in their paper. Using the neural network architecture consisting of attention pooling, we performed ten-fold cross-validation for the ‘task-failure’ classification task. The resulting performance measures are shown in Table III for comparison to FDA results.

## IV. RESULTS

### A. Classification

The results of the ten best performing models are presented in Table II. The table shows (1) which classification model structure was used; (2) which combination of inputs (functional and non-functional) were applied to that model; and (3) the resulting predictive accuracy over the dataset. All models performed better when the continuous variable ‘body mass’ was omitted. The GSAM structure generally performed best among the tested classifiers. The highest F1 score (93.5%) was achieved using $\dot{V}{\text{O}}_{2}$ functional data *and* sex and puberty covariates as predictors in a GSAM. Providing functional data alone (with no anthropometric covariates) resulted in a maximum classification F1 score of 91.1%.

After testing each individual cardiovascular signal, we constructed a ‘full’ model. This model used all functional data of HR, RR, $\dot{V}O_{2}$, and $\dot{V}{\text{CO}}_{2}$ together, along with sex, puberty level, and body mass. The results are shown in Table III. The ‘Full GSAM’ model performed best (F1 score 93.5%, accuracy 93.6%). Further, we demonstrated that the FDA method performed better than GAF and MTF encoder approaches.

### B. Functional Analysis of Variance

The statistic of interest in drawing conclusions from FANOVA was the probability of a true difference in functional means over the bootstrapped observations. A *p*-value ≤ 0.05 indicated that we could reject (*H*_0_) and conclude that a significant difference in functional means was present.

**Fig. 6.**
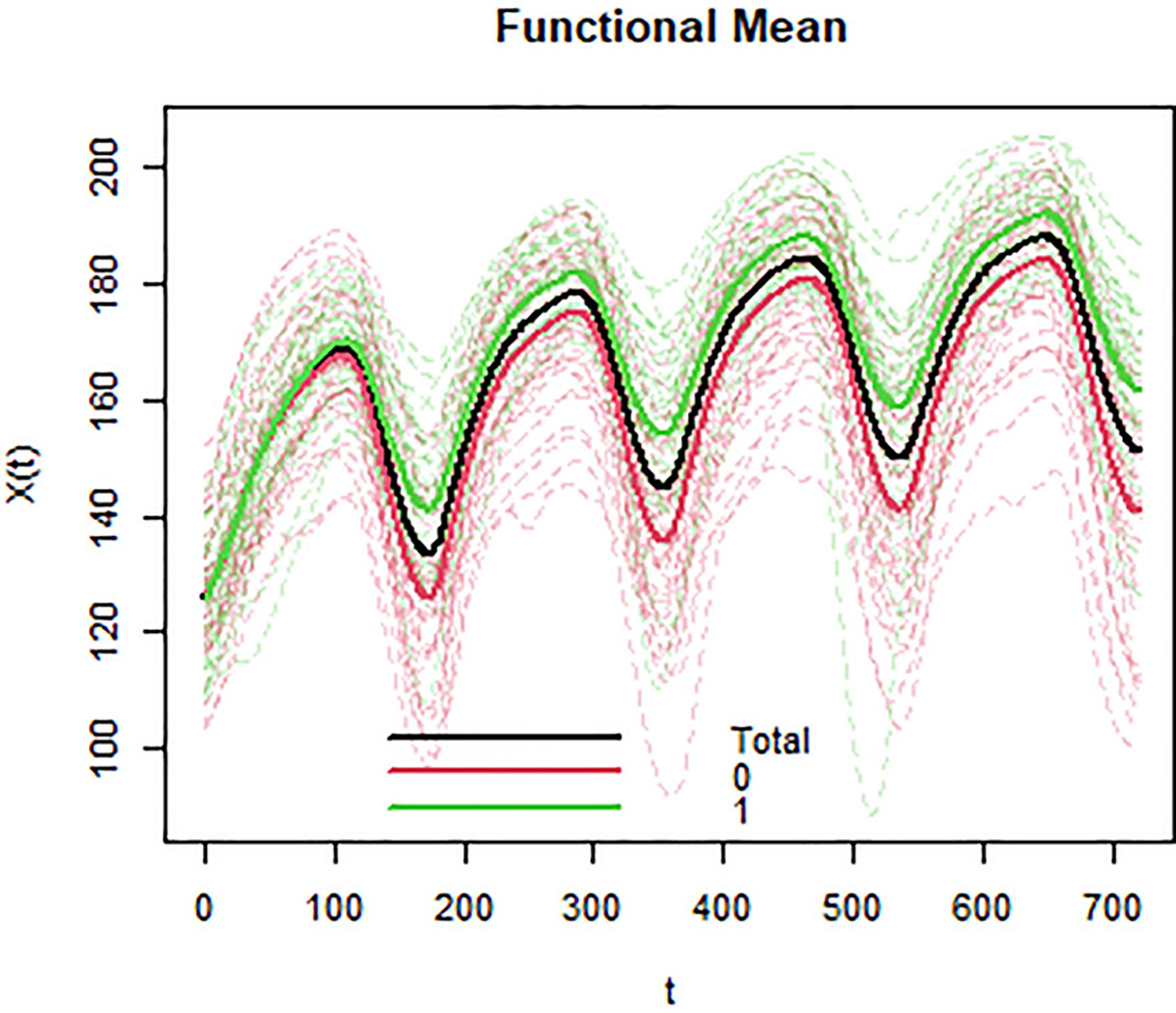
Comparison of functional means for the Heart Rate signal [X(t)] during the first four exercise bouts. Task-failures are labelled as ‘1’ with a solid green mean function. ‘Task-completers’ are labelled ‘0’ with a solid red mean function. The black line indicates the mean trajectory for all participants.

Fig. 6 is the visual depiction of HR functional means for ‘task-failures’ and ‘completers’ and compares the estimated HR curves after bootstrap resampling. FANOVA results for RR, $\dot{V}O_{2}$, and $\dot{V}{\text{CO}}_{2}$ are included in Appendix C. Table IV shows the resulting *p*-values and conclusions from FANOVA. We found that children in the ‘failure’ and ‘completer’ groups have significantly different functional means for three signals: HR, oxygen uptake rate, and carbon dioxide uptake rate. Each of these variables display higher mean functions across the four bouts for those who failed to complete the MBEB session.

## V. DISCUSSION

This is the first study to examine whether functional data analysis of breath-by-breath gas exchange and HR data could predict an individual’s ability to complete a task consisting of ten 2-min bouts of constant work rate, high-intensity exercise.

There are a few theoretical implications highlighted by our work. First, we contribute to understanding exercise-induced responses of children. The differences that we found between gender and puberty subgroups are generally in agreement with historical findings. There is evidence, for example, that healthy, early pubertal children have substantially faster HR and $\dot{V}{\text{CO}}_{2}$ exercise responses than healthy late-pubertal or adult individuals [26], [27]. $\dot{V}{\text{O}}_{2}$ kinetics appear to be less dependent on puberty status, but children typically have higher oxygen uptake per work performed than do late pubertal or adult individuals [12], [28]. These differences were identified via *t*-test after our second-by-second observations were transformed into functional data. Statistically significant functional differences between males and females were more difficult to discern in our data set and require further study.

We considered FDA’s theoretical utility in the exercise data arena. Based on model performance alone, FDA seems to be a highly useful tool for processing exercise-induced physiological signals. By transforming the raw data into appropriately smoothed functions, the outputs were quite useful for highlighting differences among the cohorts. In addition to the promising predictive capability we presented here, the general benefits of FDA were apparent. As exercise response signals are inherently noisy and non-linear (especially in younger children compared with adults), exploration of the data as smoothed functions was instrumental in our statistical analysis. Conventional statistical techniques are useful for *ramp* style exercise time series, as the on- and off-transient structure does not exist. However, these methods struggle to capture the patterns when considering multiple repeated exercise intervals.

FDA allows for handling of sparse datasets and those in which individual exercise performance intervals of the protocol are not cleanly aligned. FDA’s ability to reduce predictive error could be beneficial for exercise prescription, especially in settings where a maximal stress test is not feasible [29].

One-way functional ANOVA showed that, in general, ‘task-failures’ were characterized by a statistically significant higher functional mean HR, $\dot{V}O_{2}$, and $\dot{V}CO_{2}$ across the four bouts.

Our models included gender, maturational status, and body mass as scalar covariates alongside functional MBEB signals to identify ‘task-failures.’ $\dot{V}O_{2}$, $\dot{V}CO_{2}$, and HR were especially informative signals for predicting ‘task-failures’ based on the first four exercise bouts. Incorporating gender and puberty level was beneficial for several models. The top performing model classified ‘task-failures’ with 93.5% F1 score; by adding the anthropometric features to the functional covariate, we improved the classification rate by several points. We also showed the ability to sample from subgroups and conduct permutation *t*-tests of the functional means, testing for sex and maturational status differences. This particular comparative method is more challenging with discrete data.

With regard to the ‘body mass’ variable, the generalized spectral additive model (GSAM) that produced the best results showed that inclusion of this variable provided no additional benefit in model performance. Body mass is certainly correlated with some physiological signals. A theoretical discussion of how body mass may influence physiological and metabolic function can be found in seminal papers by A. Heusner [30], [31]. However, the degree of this correlative effect seems to be subject to a participant’s other demographics [3]. These researchers found significantly higher $\dot{V}O_{2}$, and $\dot{V}CO_{2}$, and $\dot{V}E$ costs in the early-pubertal participants for both low- and high- intensity multiple brief exercise bout (MBEB) protocols when these values were scaled to body mass. It is possible that these differences in dynamic responses between pubertal groups hindered the ability of Functional Data models to correctly predict which children would fail to complete all ten exercise bouts.

As to the practical implications of our work, FDA can also provide interpretable results for the clinician. Suppose that instead of predicting who quits exercising, we want to see the differences between healthy individuals and those with chronic disease. The graphical depictions of sub-group mean functions (Fig. 6 and Appendix C) can aid a clinician with determining whether a patient’s trajectory more closely aligns with that of a healthy or non-healthy subject. Finally, as suggested by [11], we demonstrated that meaningful medical conclusions can be drawn with measurements from shorter-duration exercise tests.

## VI. FUTURE WORK

FDA is currently a very active research topic. The performance of FDA for exercise testing on this sample of participants suggests further research opportunities. First, there exist other important frequency and gas exchange variables as calculated during CPET; work output (watts), minute ventilation $\left(\dot{V}E\right)$, respiratory quotient (RQ), and the ratio of $\dot{V}E$ to $\dot{V}CO_{2}$ ($\dot{V}E/\dot{V}CO_{2}$ slope). FDA could be applied to each of these and may prove medically useful. Some physiological signals are correlated with body mass; it would be interesting to test theories about the dynamics of gas exchange variables while specifically normalizing by lean body mass.

Further investigation is needed into the the selection of smoothing parameters and basis representation for FDA. A B-spline basis was chosen for this dataset due to the popularity and flexible nature of splines as well as the ability to capture the on- and off-transient signal patterns that resulted from MBEB. Other basis transformations should be investigated for their goodness of fit on this and other data sets. Additional analysis is also necessary to confirm that the results in this research are reproducible for the low-intensity exercise setting.

## VII. LIMITATIONS

The primary limitations of this study relate to the type of patients and quantity of exercise tests analyzed. Our participant population, while reflective of the local community at our site, was not representative of the population as a whole. Moreover, any predictive methodologies must be tested by prospective studies and analysis. Further studies will be necessary to gauge the effect of chronic disease, racial, ethnic, and other social determinants on exercise responses as children develop. The FDA method should be applied to other population groups, such as healthy young adults or pediatric patients with chronic diseases or obesity. This study’s analysis may be useful as a baseline to which we can compare the signals of diseased individuals. The inclusion of prospective data in future work should eliminate potential bias in our method of analysis.

The binary classification methods used here assume that whether or not a child completes an exercise test is an appropriate proxy for his or her physical fitness. There are undoubtedly other factors at play when a child makes the decision to quit during intense exercise. Understanding the physiological determinants that contribute to cognitive decision-making around exercise behavior will be critical for the optimal use of exercise testing in health and disease.

## Figures and Tables

**TABLE I:** Anthropometric and Peak $\dot{V}O_{2}$ in 78 Study Participants

Cohort	N	Age (yr)	Weight (kg)	Height (cm)	BMI (%tile)	Peak $\dot{V}O_{2}$ (ml/min/kg)

EPF	16	9.0 ± 1.3	28.5 ± 5.9	130.6 ± 7.4	47.3 ± 27.0	44.3 ± 7.3
EPM	20	10.7 ± 1.8	36.7 ± 11.8	143.6 ± 12.2	45.1 ± 29.9	52.3 ± 7.7
LPF	23	15.5 ± 7.8	54.8 ± 8.4	161.38 ± 6.0	54.3 ± 24.3	40.0 ± 7.9
LPM	19	16.8 ± 1.4	63.5 ± 10.3	172.8 ± 6.7	45.7 ± 25.4	54.5 ± 8.8

**TABLE II:** Individual Classification Model Performance

Model	Inputs	Accuracy (%)	F1 Score (%)

GSAM	$\dot{V}O_{2}\;+\;\text{Sex}\;+\;\text{puberty}$	93.6	93.5
GSAM	$\dot{V}O_{2}$	91.0	91.1
GSAM	$\dot{V}\text{C}O_{2}$	87.2	87.5
GSAM	$\dot{V}\text{C}O_{2}\;+\;\text{Sex}\;+\;\text{puberty}$	87.2	86.8
NNet	$\dot{V}O_{2}$	84.6	83.8
GSAM	HR + Sex + Puberty	82.1	82.1
GSAM	HR	78.2	80.5
SVM	HR	79.5	80.0
NNet	$\dot{V}\text{C}{\text{O}}_{2}$	80.8	80.0
LDA	HR	79.5	79.5

**TABLE III:** Full Classification Model Performance

Model	Accuracy (%)	F1 Score (%)

Full GSAM	93.6	93.5
Full LDA	87.2	87.2
Full RPART	83.3	84.0
Full SVM	77.0	78.0
Full NNet	70.5	72.3
Full KNN	65.4	69.0
Full RandomForest	66.7	66.7

GADF + Attention	80.8	80.0
GASF + Attention	76.9	76.9
MTF + Attention	74.4	73.0

**TABLE IV:** FANOVA Results

Response Variable	p-value	Conclusion

Heart Rate	0.000	S.S. difference in means
Respiratory Rate	0.186	not S.S. difference in means
$\dot{V}O_{2}$	0.000	S.S. difference in means
$\dot{V}CO_{2}$	0.000	S.S. difference in means

**Fig. 7.**
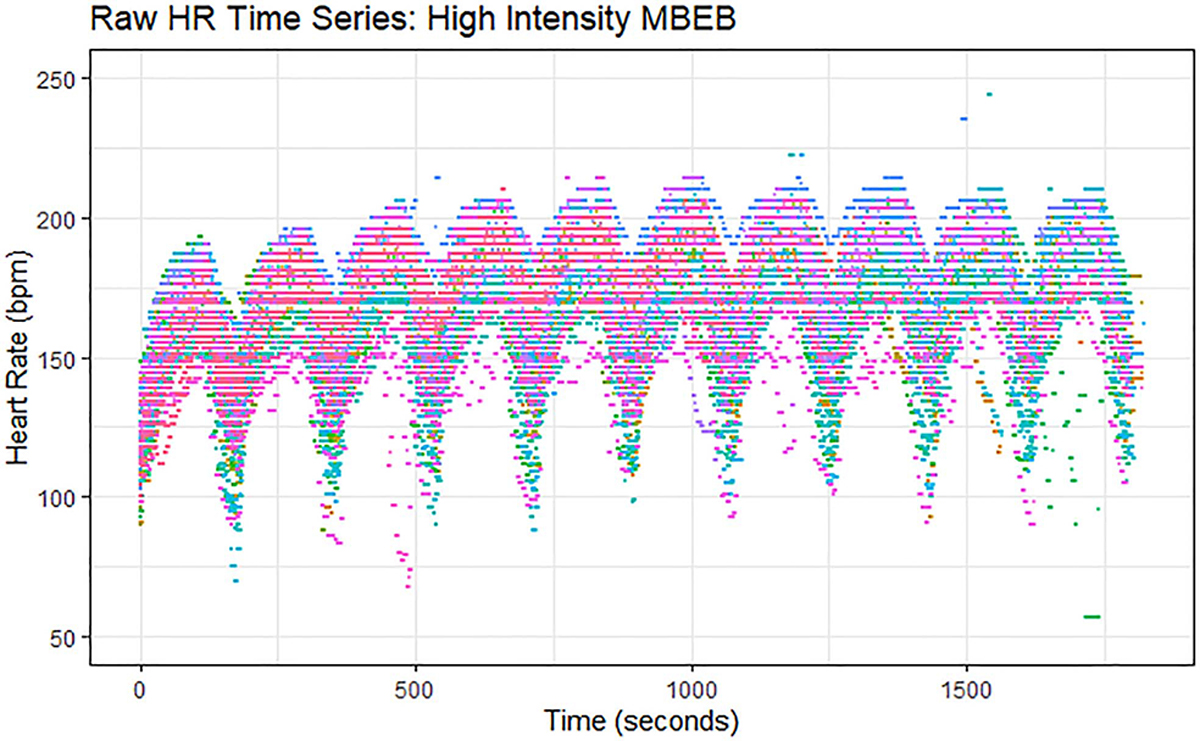
Ten bouts of Heart Rate data, originally observed breath-by-breath and time interpolated to a second-by-second representation. Each participant’s observations are shown with a unique color.

**Fig. 8.**
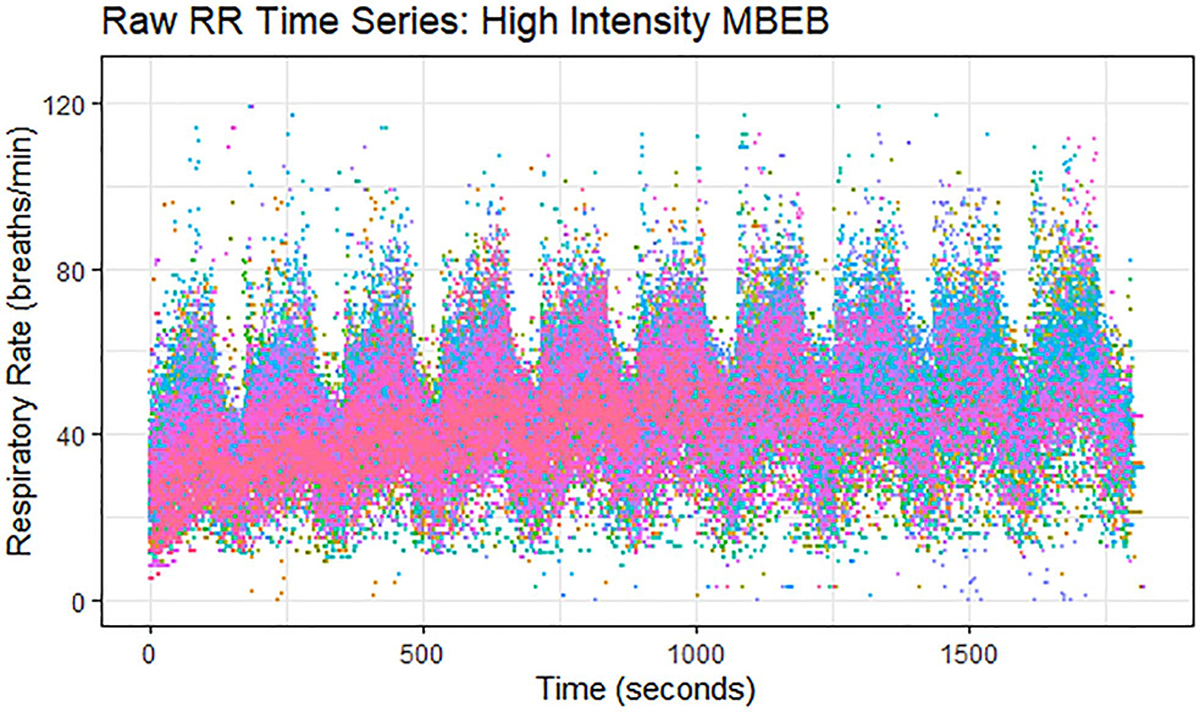
Ten bouts of Respiratory Rate data, originally observed breath-by-breath and time interpolated to a second-by-second representation.

**Fig. 9.**
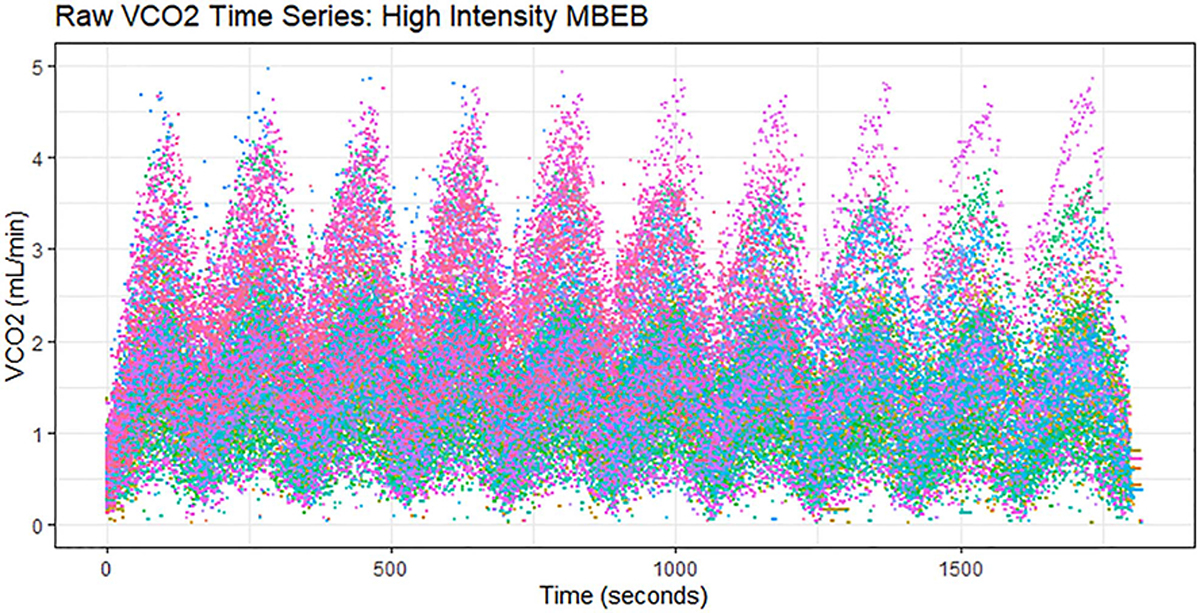
Ten bouts of O_2_ Uptake Rate data, originally observed breath-by-breath and time interpolated to a second-by-second representation.

**Fig. 10.**
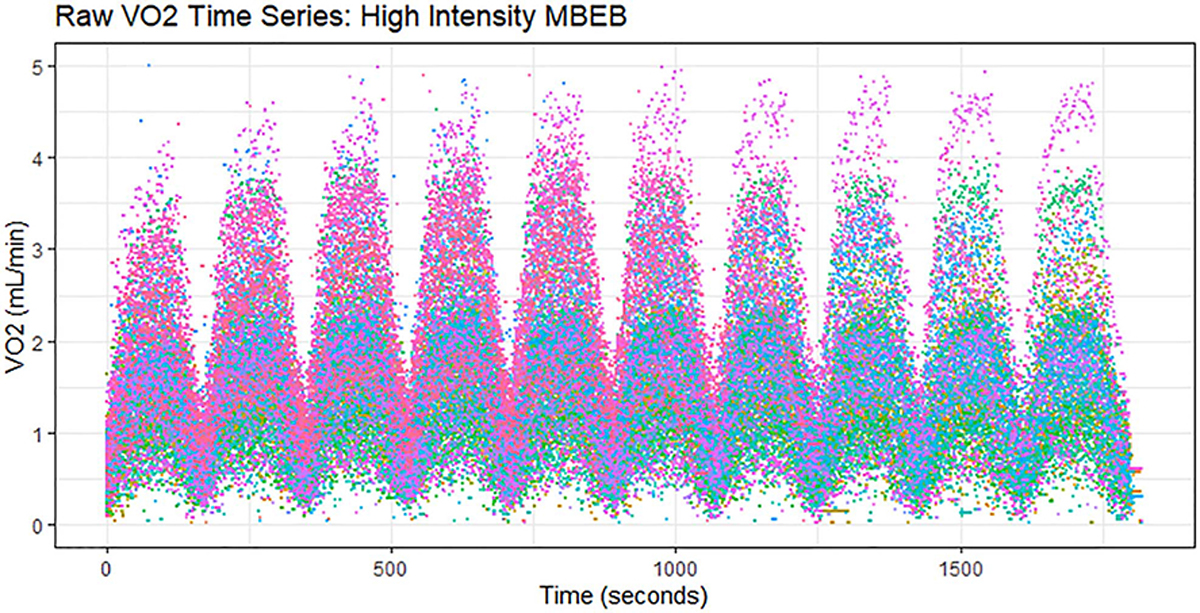
Ten bouts of CO_2_ Uptake Rate data, originally observed breath-by-breath and time interpolated to a second-by-second representation.

**Fig. 11.**
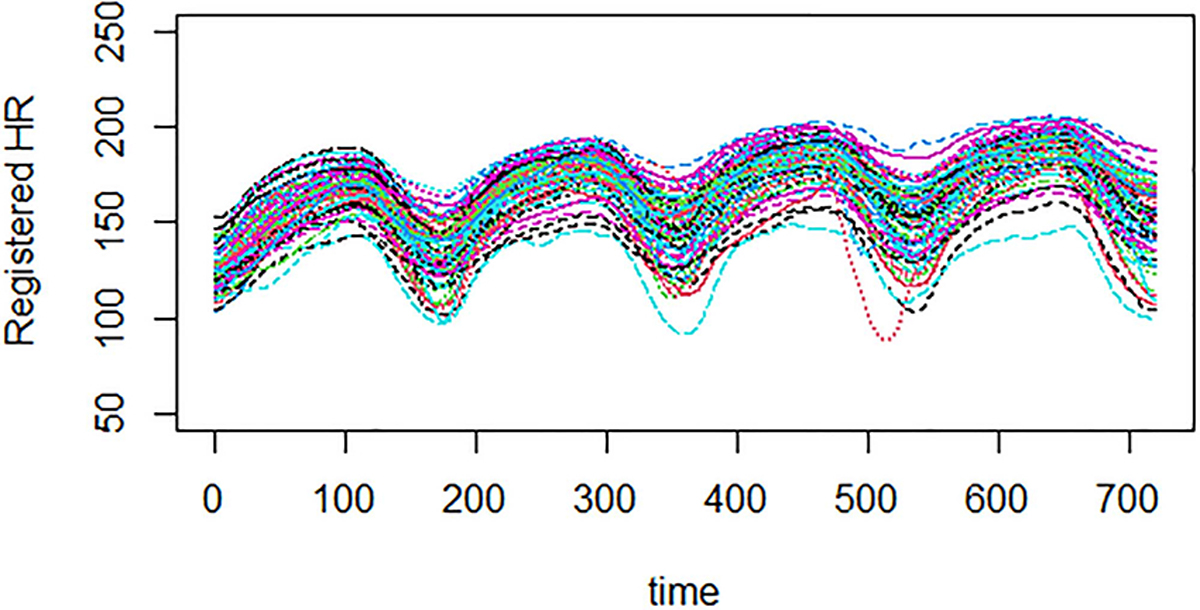
Four bouts of Heart Rate after converting the discrete time series to 78 smoothed and continuously registered functional data objects.

**Fig. 12.**
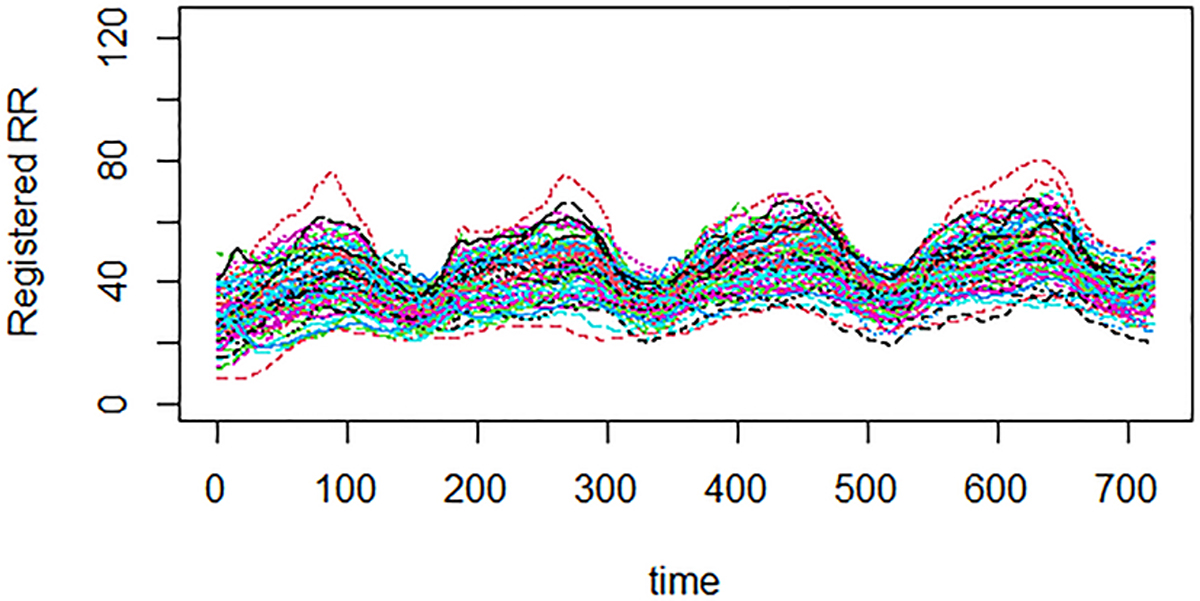
Four bouts of Respiratory Rate after converting the discrete time series to 78 smoothed and continuously registered functional data objects.

**Fig. 13.**
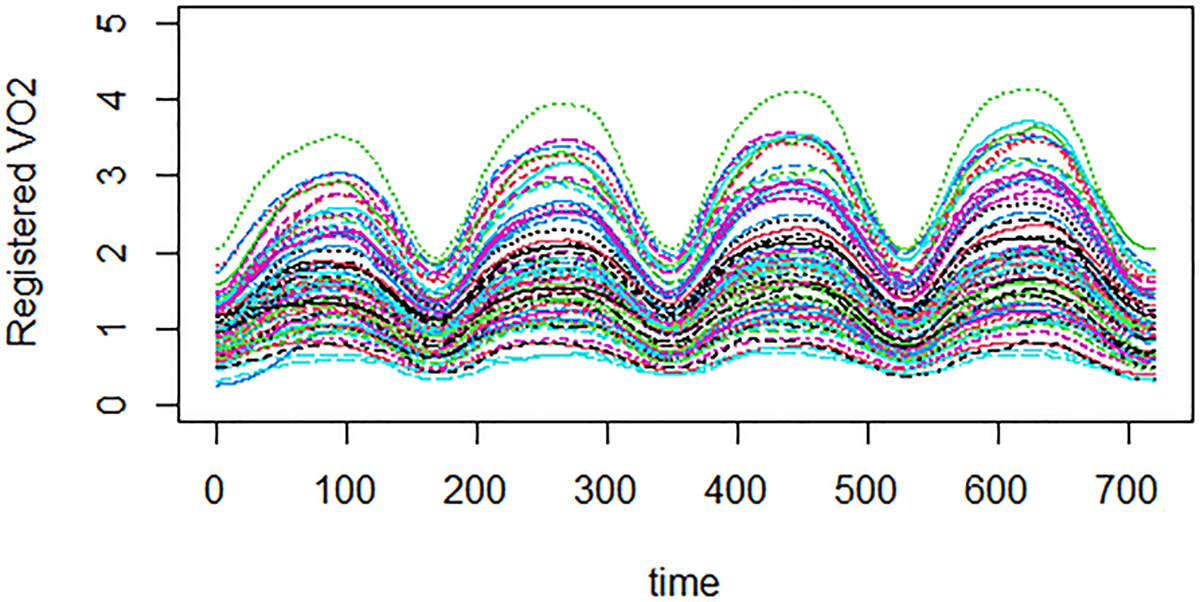
Four bouts of O_2_ uptake rate after converting the discrete time series to 78 smoothed and continuously registered functional data objects.

**Fig. 14.**
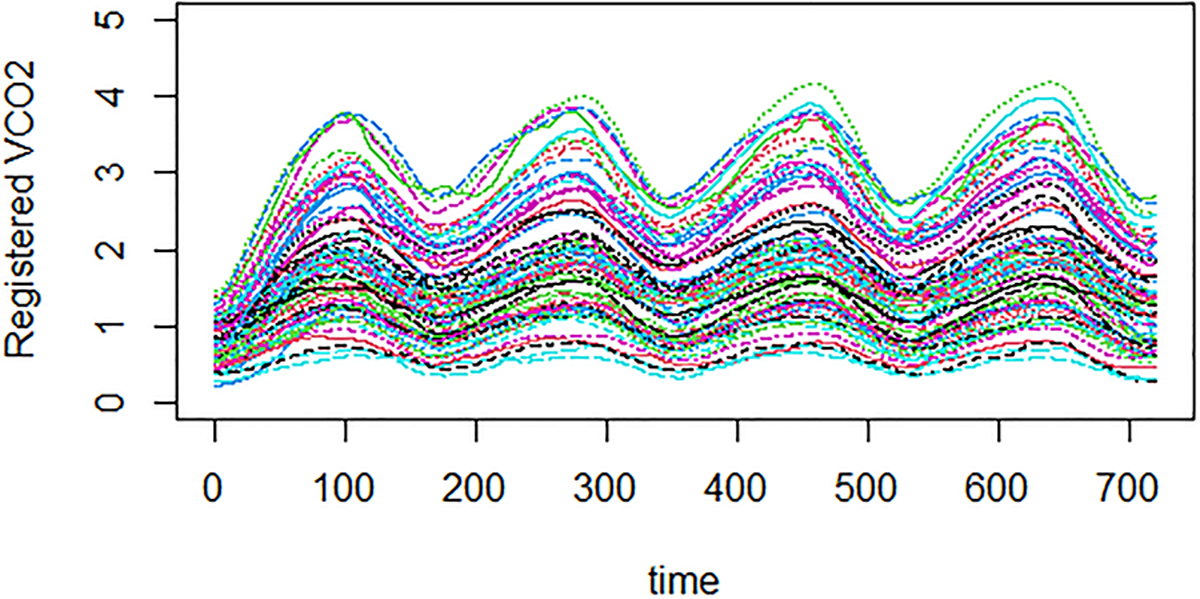
Four bouts of CO_2_ uptake rate after converting the discrete time series to 78 smoothed and continuously registered functional data objects.

**Fig. 15.**
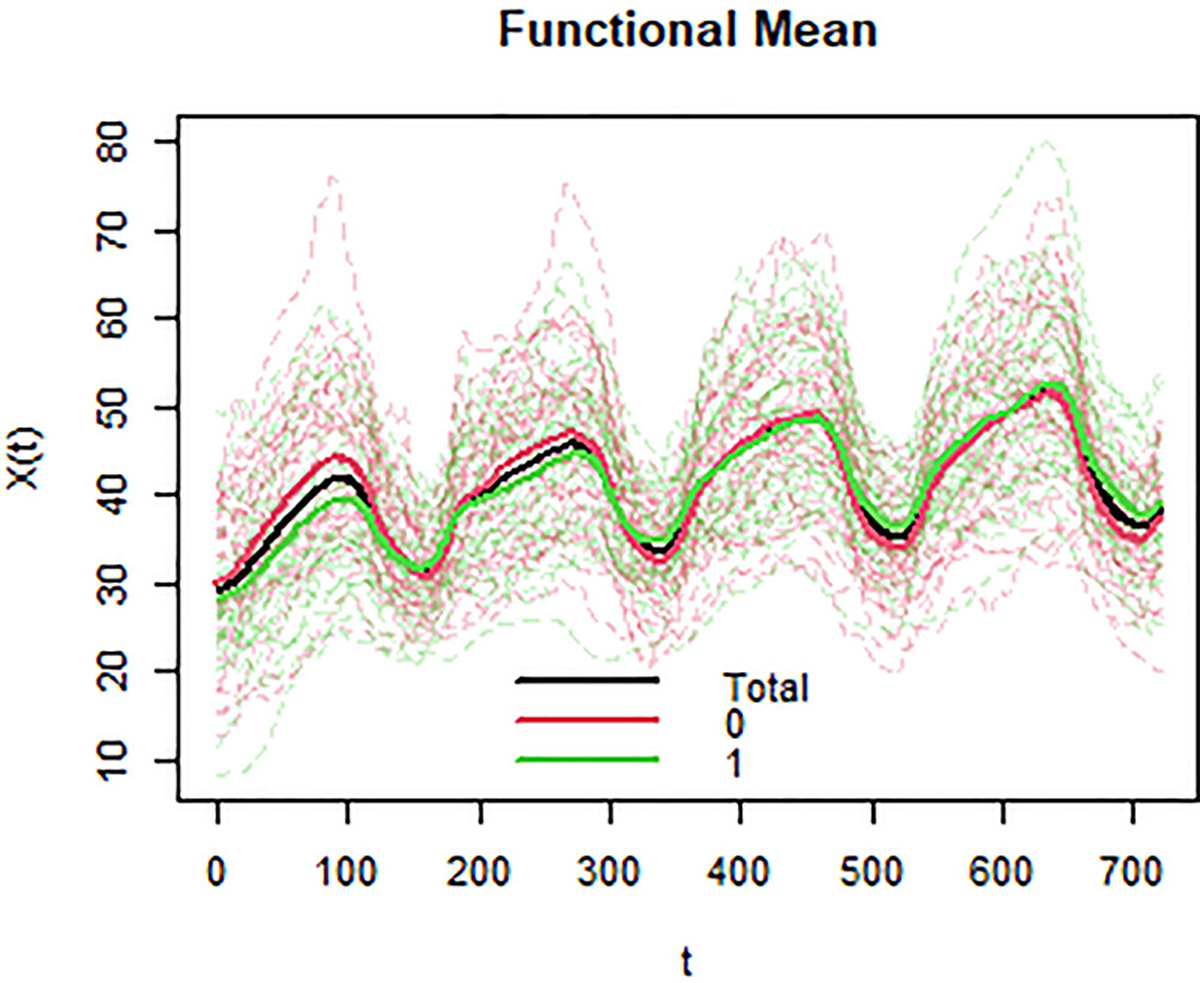
Comparison of functional means for the RR signal during the first four exercise bouts. *p*-value = **0.186**. Participants who failed to complete ten bouts during MBEB are labelled as ‘1’ and colored green. The red line depicts the functional mean for ‘task-completers.’ The black line indicates the mean trajectory for all 78 participants. The plot on the right shows Heart Rate curves for MBEB ‘task-failures’ (green) and ‘task-completers’ (red), bootstrapped 500 times. The black line indicates the mean trajectory for all 78 participants. Notice that there is substantial overlap between the two groups’ signals; quitters and non-quitters have virtually indistinguishable respiratory rates.

**Fig. 16.**
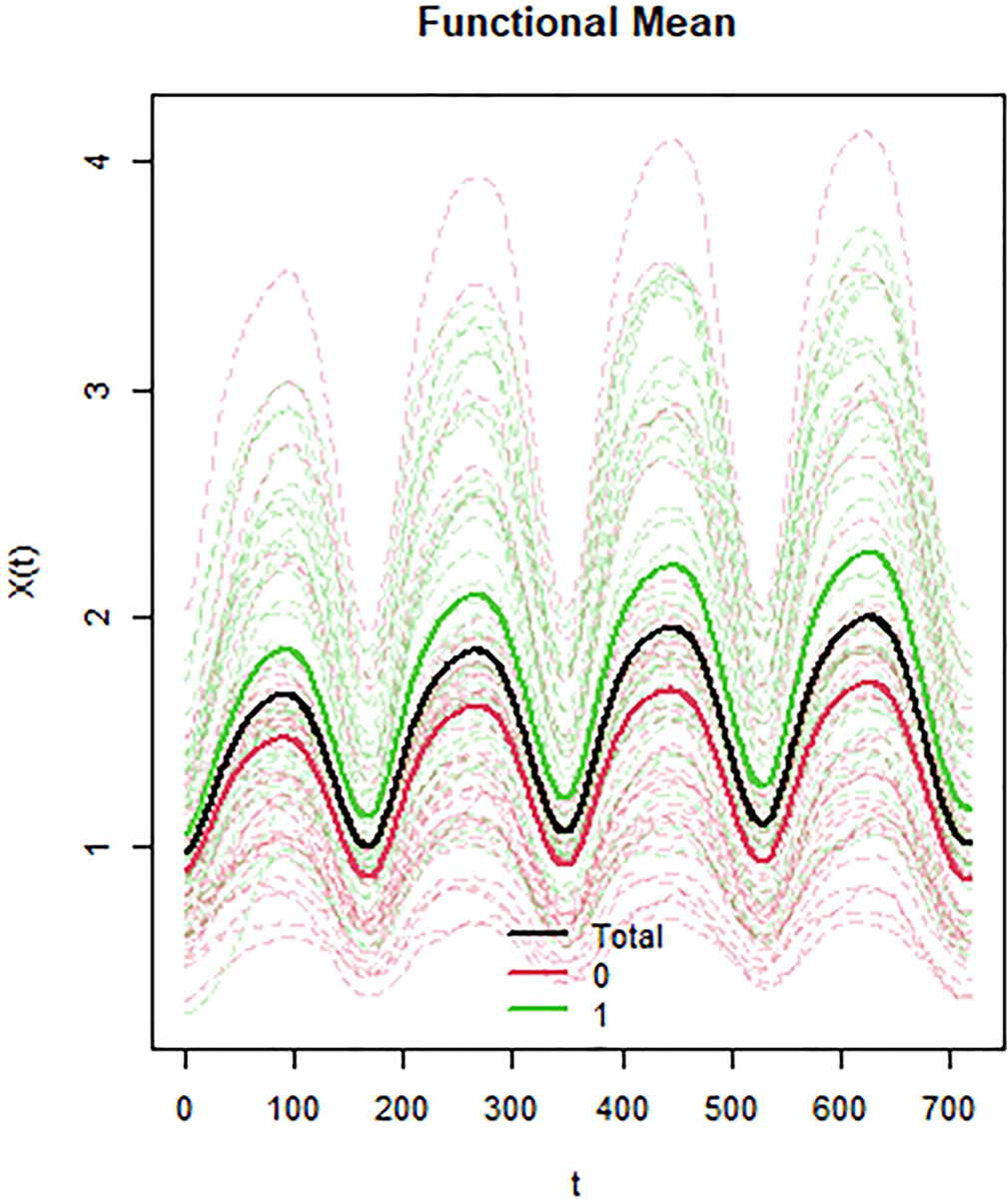
Comparison of functional means for the $\dot{V}{\text{O}}_{2}$ signal during the first four exercise bouts. *p*-value = **0**. Participants who failed to complete ten bouts during MBEB are labelled as ‘1’ and colored green. The red line depicts the functional mean for ‘task-completers.’ The black line indicates the mean trajectory for all 78 participants. The plot on the right shows Heart Rate curves for MBEB ‘task-failures’ (green) and ‘task-completers’ (red), bootstrapped 500 times. The black line represents the bootstrapped mean function for 78 participants.

**Fig. 17.**
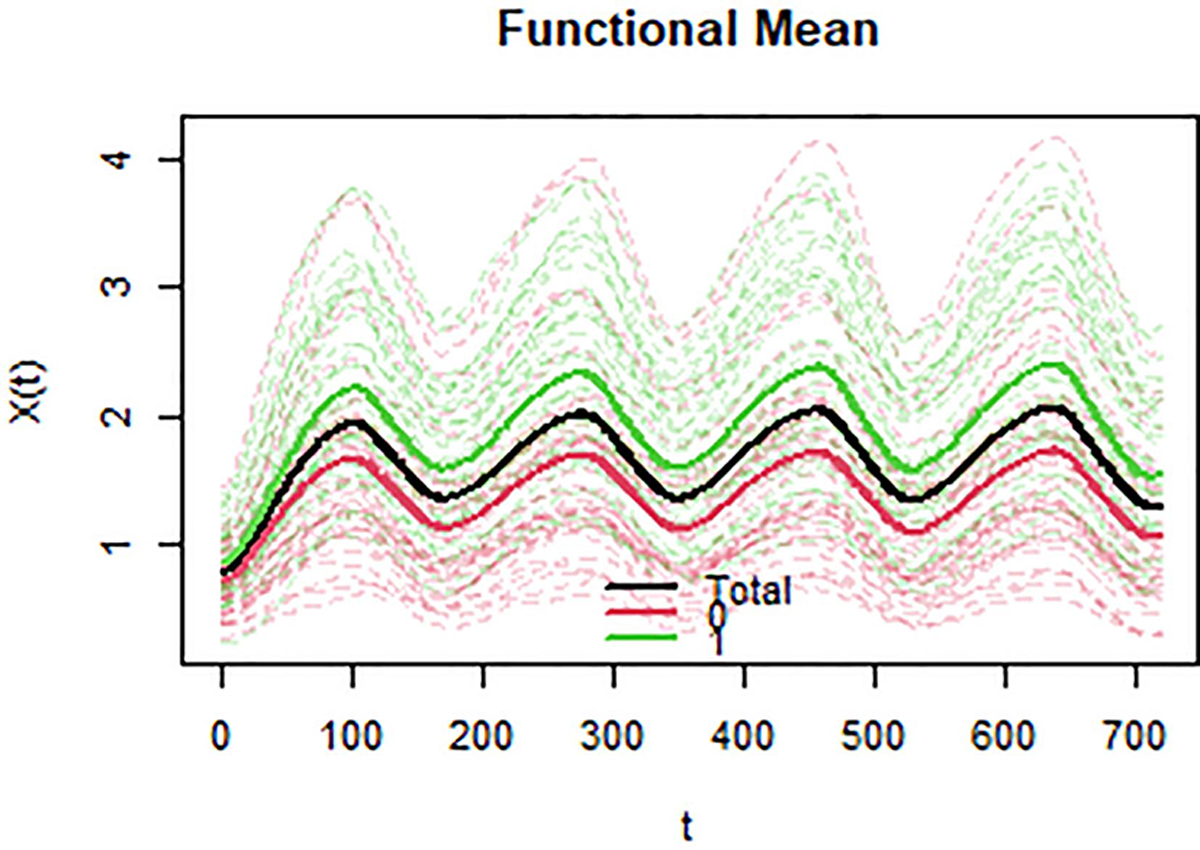
Comparison of functional means for the $\dot{V}{\text{CO}}_{2}$ signal during the first four exercise bouts. *p*-value = **0**. Participants who failed to complete ten bouts during MBEB are labelled as ‘1’ and colored green. The red line depicts the functional mean for ‘task-completers.’ The black line indicates the mean trajectory for all 78 participants. The plot on the right shows Heart Rate curves for MBEB ‘task-failures’ (green) and ‘task-completers’ (red), bootstrapped 500 times. The black line represents the bootstrapped mean function for 78 participants.

## APPENDIX A RAW DATA PLOTS

The figures below are the second-by-second observations of our four variables of interest: HR, RR, $\dot{V}O_{2}$, and $\dot{V}CO_{2}$.

## APPENDIX B SMOOTHED & REGISTERED DATA PLOTS

The figures below are the functional data representations of our four variables of interest: HR, RR, $\dot{V}O_{2}$, and $\dot{V}CO_{2}$.

## APPENDIX C FANOVA RESULTS

Functional ANOVA results for the RR, $\dot{V}{\text{O}}_{2}$, and $\dot{V}{\text{CO}}_{2}$ guided the investigation of the null hypothesis. For *p*-values ≤ 0.05, we reject the null hypothesis and conclude that there is a statistically significant difference in the functional means for ‘task-failures’ and ‘task-completers.’

## APPENDIX D CLASSIFICATION MODEL DESCRIPTIONS

This appendix details the structure of each classification model in the Functional Data approach. Models were built with consistent parameters to allow for performance comparison. Note that the individual models use only functional data from **one** physiological signal, and the multivariate models use functional data coefficients from **all four** signals. Also, the full multivariate models include *BodyMass* as a third anthropometric scalar variable.

All modeling was performed in Rstudio (Version 1.4.1103). FDA was conducted in R using the ‘fda’ package (version 5.5.0) [23] and the ‘fda.usc’ package (version 2.0.2) [25]. Wrapper versions of the following packages were called within the ‘fda.usc’ functions:

- RPART: rpart package
- RandomForest: randomForest package
- SVM: e1071 package
- LDA: MASS package
- Neural Network: nnet package

The binary class ‘quit’ (1 or 0) was predicted with the following covariates (*X*_[0,720]_ represents the response variable and the function *s*(·) denotes an additive effect over the variable):

- GSAM: *s*(*X*_[0_,720])

- equal weights (1) were used for all observations in GSAM models
- The probability value for binary discriminant (i.e. classification threshold) was optimized within each GSAM model; we searched across a range between 0.3 and 0.8, and the threshold which produced the highest F1 score was selected.
  - GSAM + Covariates: *s*(*X*_[0,720]_) + *Gender* + *PubertyLevel* (+ *BodyMass* for the full model)
  - RPART: *s*(*X*_[0,720]_) + *Gender* + *PubertyLevel* (+ *BodyMass* for the full model)
- the value of prior probabilities was set to the default for rpart
  - K-Nearest Neighbors: *X*[0,720] + *Gender* + *PubertyLevel* (+ *BodyMass* for the full model)
- the *k* number of nearest neighbors was chosen based on trial and error, to determine which *k* resulted in the lowest classification error. Therefore, k varies between 12 and 14 among the models.
  - RandomForest: *X*_[0,720]_ + *Gender* + *PubertyLevel* (+ *BodyMass* for the full model)
- we used the default value for the number of trees to grow (500) and the number of variables available for splitting at each tree node (square root of total number of variables)
  - Support Vector Machines: *X*_[0,720]_ + *Gender* + *PubertyLevel* (+ *BodyMass* for the full model)
- default values were used for the C parameter (1) and *γ* parameter (1/data dimension) in the radial basis function kernel
  - Linear Discriminant Analysis: *X*_[0,720]_ + *Gender* + *PubertyLevel* (+ *BodyMass* for the full multivariate model)
- the important parameter was the prior probabilities of class membership; with our balanced data, we used the class proportions for the training set
  - Neural Network: *X*_[0,720]_ + *Gender* + *PubertyLevel* (+ *BodyMass* for the full model)
- we used the default value for weights (1) in the neural net
